# Long-acting Inhaled Bronchodilator and Risk of Vascular Events in Patients With Chronic Obstructive Pulmonary Disease in Taiwan Population

**DOI:** 10.1097/MD.0000000000002306

**Published:** 2015-12-28

**Authors:** Ming-Jun Tsai, Chung-Yu Chen, Yaw-Bin Huang, Hsiao-Chung Chao, Chih-Jen Yang, Pei-Jin Lin, Yi-Hung Tsai

**Affiliations:** From the Department of Neurology, China Medical University Hospital (M-JT); School of Medicine, Medical College, China Medical University, Taichung (M-JT); Department of Neurology, China Medical University An-Nan Hospital, Tainan (M-JT); Department of Pharmacy, Kaohsiung Medical University Hospital (C-YC, Y-BH); School of Pharmacy, Master Program in Clinical Pharmacy, Kaohsiung Medical University, Kaohsiung (C-YC, Y-BH, P-JL, Y-HT); Department of Pharmacy, Chi Mei Medical Center, Tainan (H-CC); Department of Respiratory Therapy, Kaohsiung Medical University, Kaohsiung (C-JY); and Department of Pharmacy, Kaohsiung Veterans General Hospital, Kaohsiung, Taiwan, ROC (P-JL).

## Abstract

A combination of long-acting anticholinergic agents (LAACs) and long-acting β_2_-adrenergic receptor agonist (LABA) is effective in improving lung function in chronic obstructive pulmonary disease (COPD) compared with monotherapy. However, evidence on whether this combination increases the incidence of stroke or other cardiac events remains sparse. The objective of the present study was to investigate the incidence of stroke and other cardiovascular diseases in COPD patients treated with LAAC, LABA, or a combination of the 2.

We conducted this population-based study using the Taiwan National Health Insurance Research Database (1997–2008), identifying COPD patients and their prescribed medication from the International Classification of Disease, Ninth Revision codes 490–492 or 496. A multivariate Cox proportional-hazards model was used to compare the risk of stroke and other cardiovascular diseases over the 11-year period after treatment with LAAC or LABA only or in combination.

Of the 596 COPD patients (mean age 70 y), 196 were treated with LAAC, 318 with LABA, and 82 were treated with a combination. The overall incidence of stroke (8.53%) significantly increased in the combination group compared with LAAC (2.04%) or LABA (1.26%) only. In the Cox regression analysis, the adjusted hazard ratio over the 11-year survey period for stroke in patients treated with the combination compared with LABA only was 1.04 (95% confidence interval, 1.06–2.99) and for LAAC, it was 0.31 (95% confidence interval, 0.02–2.32).

This cohort study using a large health insurance database showed that treating patients with COPD, with a combination of LAAC and LABA, may be associated with an increased hazard of stroke compared with treatment with either agent alone. We should be particularly cautious about comedication of LAAC and LABA in patients with COPD.

## INTRODUCTION

Chronic obstructive pulmonary disease (COPD) is a major cause of chronic morbidity and mortality worldwide. Bronchodilators are the primary treatment for COPD and include anticholinergics, β_2_-agonists, methylxanthines, and corticosteroids used alone or in combination.^1^ The effectiveness and safety of long-acting anticholinergic agents (LAACs) and long-acting β_2_-adrenergic receptor agonists (LABAs) have been described in meta-analyses^2,3^ or retrospective cohort studies,^4,5^ reporting an imprecise reduction in mortality and a significantly lower risk for COPD exacerbations. Accumulated evidence has shown that a combination of LAAC and LABA is effective in improving lung function tests and quality of life compared with monotherapy in patients with COPD, particularly when the COPD is severe.^6–9^

However, from previous studies, there has been concern that LAAC and LABA drugs may increase hazard of cardiovascular and/or cardio-cerebrovascular complications, including heart failure, angina cardiac dysrhythmias, myocardial infarction, or stroke.^10–13^ In contrast, the results of the clinical trial and previous observational studies showed that the incident rate and hazard of stroke or cardiac events were not significantly different between LAAC and LABA.^14–17^ Therefore, current evidences indicated cardiovascular or cardio-cerebrovascular safety is a matter of debate in patients with COPD using LAAC or LABA. In particular, it remains unclear whether a combination of LAAC with LABA or LABA with inhaled corticosteroids (ICS) increases the incidence of stroke or other cardiac events.^17–19^

The purpose of the present study was to compare, using the Taiwan National Health Insurance (NHI) Database, the hazard of stroke and other cardiovascular diseases arising from combination therapies LAAC/LABA or LABA/ICS with monotherapies of LABA or LAAC only in patients with COPD.

## METHODS AND MATERIALS

### Source of Data

This population-based retrospective cohort study was based on the Longitudinal Health Insurance Database (LHID) 2005, which included 1 million individuals randomly sampled from the 2005 Registry for Beneficiaries of Taiwan's National Health Insurance Research Database (NHIRD). This database, provided by the National Health Research Institute, included data on outpatients, hospital inpatient care, ambulatory care, dental services and prescription drugs. In 1995, NHI, a government-run insurer with a single-payer insurance system, was established in Taiwan to ensure the health of the entire nation and to prevent social problems caused by poverty and diseases. By December 2008, 22.918 million individuals were enrolled in the program nationwide, with a coverage rate of 99.5% in Taiwan. There was no significant difference in sex distribution (*χ*^2^ = 0.008, df = 1, *P* = 0.931) between the patients in LHID 2005 and those in the original NHIRD. This study was approved by the Institutional Review Board of Kaohsiung Medical University Hospital on February 24, 2015 (KMUH-IRB-EXEMPT-20150009). However, this retrospective descriptive study does not require patient consent according to the current rules in our Hospital and NHIRD.

### Study Design

A cohort of adults diagnosed between 1997 and 2008 with first-time COPD (*International Classification of Disease, Ninth Revision* [ICD-9] codes: 490–492, 496; A-code: A323 and A325) was identified from LHID 2005. Otherwise, patients with COPD were included only if they had been diagnosed twice in our surveys. We excluded patients diagnosed with asthma (ICD-9: 493), pulmonary hypertension (ICD-9: 416.0), sleep apnea (ICD-9: 327.23, 780.57), pneumonia (ICD-9: 486, 507.0–507.8), or who were <18 years old at the date of first-time COPD diagnosis (which we took as the index date in our study). We also excluded patients diagnosed with stroke (ICD-9: 430–438), heart failure (ICD-9: 428), ventricular arrhythmia (ICD-9: 427, 785.0, 785.1), myocardial infarction (ICD-9: 410), or angina pectoris (ICD-9: 413) before the index date.

We divided the population of COPD patients into 3 groups according to the drug treatment received: LAAC only, LABA only, or a combination (LAAC/LABA or LABA/ICS). We selected the patients who were prescribed LAAC (tiotropium), LABA (salmeterol or formoterol), or a combination (salmeterol/fluticasone or formoterol/budesonide) between 1997 and 2008, and who had used either LAAC or LABA or LABA/ICS continuously over 1 year. The medication included had the following anatomical therapeutic chemical classifications: tiotropium handihaler (R03BB04), salmeterol (R03AC12), formoterol (R03AC13), salmeterol/fluticasone (R03AK06), and formoterol/budesonide (R03AK07). To decreasing selection bias due to LABA was approved using in NHI earlier than LAAC or LABA/ICS, LABA group was selected as the control group in this study (Table 1).

**TABLE 1:**
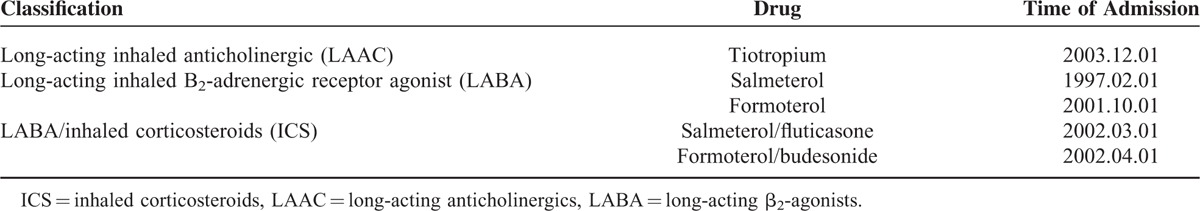
Admission of Long-acting Inhaled Bronchodilator by Taiwan Food and Drug Administration (TFDA)

Patients were followed from index date until one of the following events occurred: diagnosis of stroke, other cardiac event or death occurred, December 31, 2008 was reached, or the patient withdrew from the NHI. The baseline history of comorbidities was identified by diagnostic codes from one outpatient diagnosis or inpatient diagnosis before the index date. Comorbidities included hypertension (ICD-9: 401–405), diabetes mellitus (ICD-9: 250), hyperlipidemia (ICD-9: 272), and tuberculosis (ICD-9: 010.90). In addition, we evaluated demographical characteristics for patients with the different treatments, including sex and age. The use of concomitant drugs that might influence the efficacy of LAAC or LABA was identified according to the prescriptions within 1 year before index date. These drugs included LAAC, LABA, short-acting anticholinergics, short-acting β_2_-adrenergic receptor agonists, ICS, xanthenes, or oral steroid medication.

### Outcomes

The main outcomes evaluated were admission to hospital for stroke, heart failure, ventricular arrhythmia, myocardial infarction, or angina pectoris, based on the primary or secondary diagnoses (ICD-9). The incidence was calculated for each disease outcome. In addition, the secondary outcomes were combined into 2 composite endpoints: cardiovascular events and cardio-cerebrovascular events. Cardiovascular events included heart failure, ventricular arrhythmia, myocardial infarction, and angina pectoris. Cardio-cerebrovascular events included the cardiovascular events and stroke.

### Statistics

The hazard of outcomes during the 11-year follow-up was investigated for each treatment: LAAC or LABA only or a combination. Continuous and categorical variables were compared between each group with Student *t* test and analysis of variance (ANOVA), as appropriate. A post hoc Scheffe analysis was used. All data were expressed as frequency (percentage) and mean ± standard deviation (SD). The incidence of outcomes with the different treatments was calculated using the total number of outcomes that occurred for each disease from 1997 to 2008 as numerator and the total number of cases of each disease as denominator. The onset time of outcomes was calculated for the different treatments as the average duration from index date to first-time diagnosis of the outcome in the 11-year survey. A Cox proportional-hazards model was used to assess the risk of each outcome during the follow-up period for the LAAC only or combination treatment groups compared with that for the LABA-only group. The multivariable model was adjusted for age, sex, comorbidities, and comedication. Analyses and calculations were performed using SAS ver. 9.3 (SAS Institute, Inc., Cary, NC), and statistical significance was inferred at a 2-sided *P* value of <0.05.

## RESULTS

### Characterization of the Study Participants in 3 Groups

**FIGURE 1:**
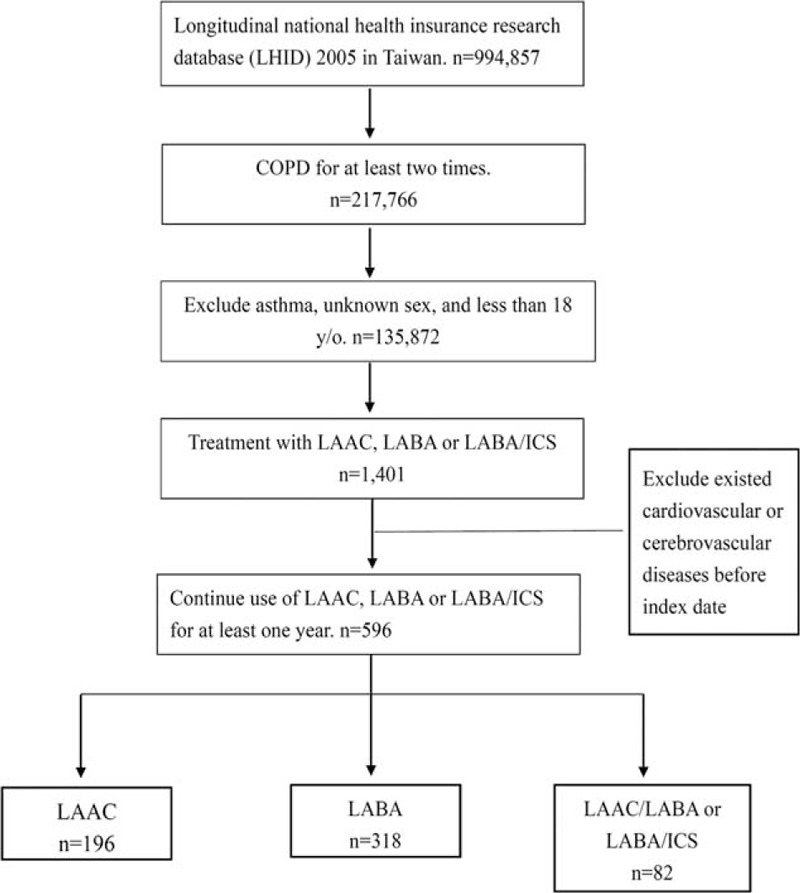
Flow chart of patient with COPD derived from longitudinal national health insurance research database. COPD = chronic obstructive pulmonary disease.

In total, 596 patients used at least one of LAAC (n = 196), LABA (n = 318), LAAC/LABA, or LABA/ ICS (n = 82) continuously for at least 1 year, as shown in Figure 1. Demographic information including age, sex, comorbidity, and medication use is shown in Table 2. There were no statistically significant differences between the 3 groups in age, sex, and comorbidity, with a mean age of about 70 and a male predominance (about 86%) in all the groups.

**TABLE 2:**
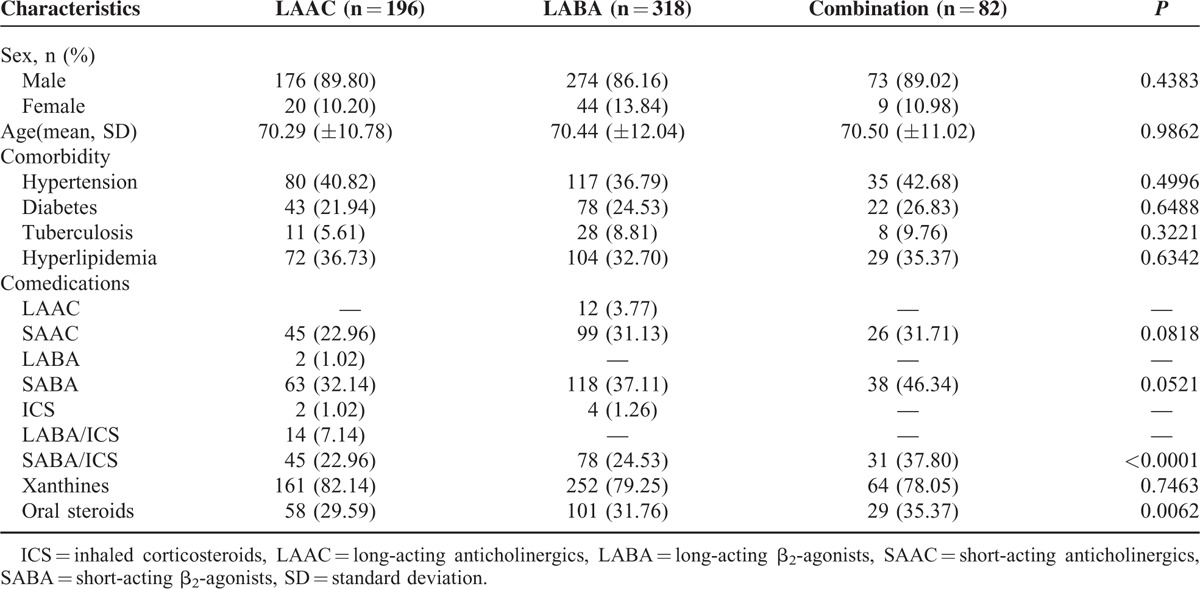
Demographic Characteristics, Comorbidity, and Comedication of Chronic Obstructive Pulmonary Disease in the Retrospective Cohort Study

### Incidence and Onset Time of Cardiac Failure, Arrhythmia, Myocardial Infarction, Angina, or Stroke in the 3 Groups

The incidences of different vascular events among the patients with COPD after more than a year of taking LAAC, LABA, and combination are shown in Table 3. From the long-term survey, there was no statistically significant difference between the 3 groups of COPD patients in developing cardiovascular diseases. However, the incident rates of stroke over the 11-year survey in the LAAC only, LABA only, and combination groups were 2.04%, 1.26%, and 8.53%, respectively—a difference that was statistically significant (*P* = 0.0033).

**TABLE 3:**
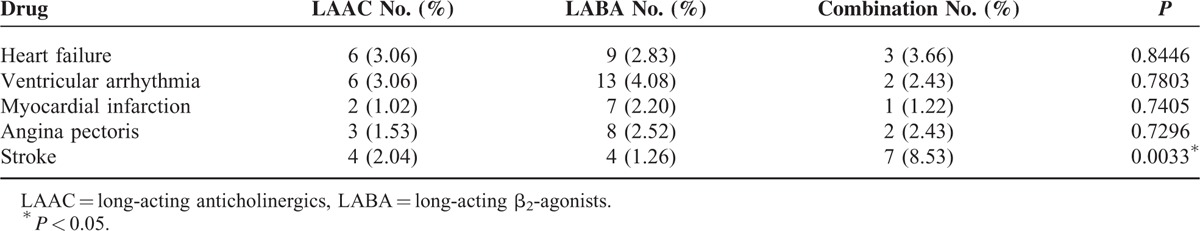
Cumulative Incidence of Cardiovascular or Cerebrovascular Events Between Inhaled Bronchodilators in Follow-up Period Stratified by LAAC Only, LABA Only, and Combination (LAAC/LABA or LABA/ICS)

Table 4 shows that the mean time to onset of cardiovascular events was shortest for the combination group and longest for the LABA-only group, although these differences were not statistically significant. However, the mean time to onset of strokes did differ significantly between the groups, being 898.39 ± 9.6 days for the LAAC-only group, 1495.20 ± 9.06 days for the LABA-only group, and 1147.52 ± 4.26 days for the combination group (*P* < 0.0001). Scheffe post hoc test also showed that patients in the LAAC group had shorter onset time of strokes than those in the LABA-only or combination group.

**TABLE 4:**
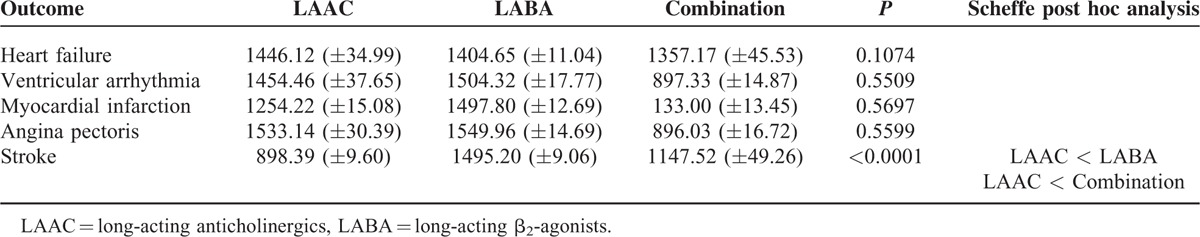
Comparisons of the Onset Time (d) of Outcome Between Inhaled Bronchodilators After Administration of Drugs, Stratified by LAAC Only, LABA Only, and Combination (LAAC/LABA or LABA/ICS)

### Hazard Ratio of Outcomes Comparing the Different Treatment Groups

Comparing the LAAC-only group with the LABA-only group, the observed crude hazard ratio (cHR) for cardiovascular events was 0.55 (95% confidence interval [CI], 0.21–1.06) and adjusted hazard ratio (aHR) was 0.49 (95% CI, 0.14–1.01). A similar trend was seen for cardio-cerebrovascular events or each outcomes comparing the LAAC-only and LABA-only groups, as shown in Table 5.

**TABLE 5:**
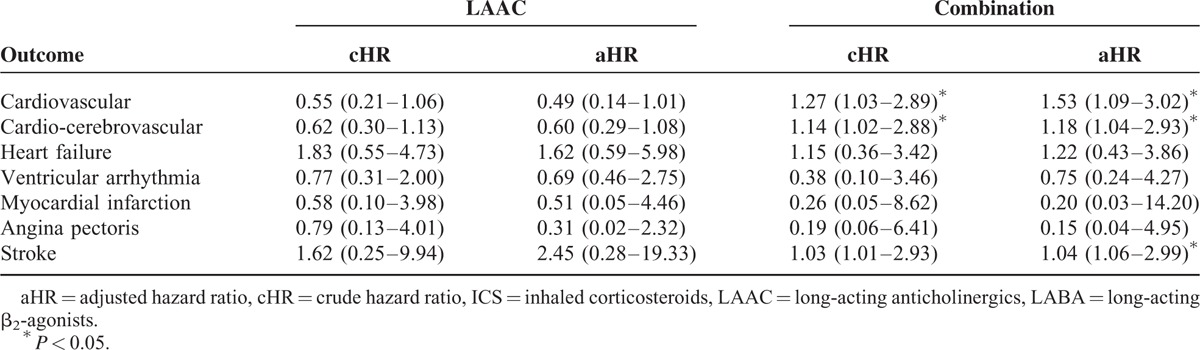
Comparisons of the Hazard of Vascular Disease Between LAAC Only and Combination (LAAC/LABA or LABA/ICS) With LABA Only

Comparing the combination group with the LABA-only group showed an increased hazard of cardiovascular events: cHR 1.27 (95% CI, 1.03–2.89) and aHR 1.53 (95% CI, 1.03–2.89). Cardio-cerebrovascular events also showed increased hazard: cHR 1.14 (95% CI, 1.02–2.88) and aHR 1.18 (95% CI, 1.04–2.93). In particular, the risk of stroke was greater in the combination group: cHR 1.03 (95% CI, 1.01–2.93) and aHR 1.04 (95% CI, 1.06–2.99).

## DISCUSSION

The present study mainly found LAAC/LABA or LABA/ICS was associated with an increased risk of cardiovascular events or stroke than with monotherapies of LABA or LAAC only in patients with COPD. Previous cohort studies had reported risks of vascular diseases of treatment with LABA or LAAC only in COPD.^4,11,18,20,21^ Wang et al^22^ reported that ipratropium had an increased risk of stroke than nonusers in COPD patients, with populations similar to that of the present study. However, the design of our study included 596 COPD patients without other pulmonary diseases, and we subdivided our population into 3 groups (LAAC-only users, LABA-only users, and patients receiving a combination of LAAC/LABA or LABA/ICS) to evaluate the risk of outcome.

The results showed that the risks of cardiovascular events or stroke were higher with the combination treatment than with LABA only, but they were not observed for LAAC-only than LABA-only group. These results were similar to those of Rodrigo et al, who reported that LAAC did not increase the risk of cardiovascular and cerebrovascular events compared with LABA.^23^ Nevertheless, from previous research, there was limited information about evaluating hazards of cardiovascular and cerebrovascular events between ICS/LABA combination and LABA or LAAC only. Otherwise, recent meta-analysis and researches have shown that combination of umeclidinium and vilanterol or indacaterol and glycopyrronium may not increase the risk of cardiovascular or other serious side effects.^9,24,25^ This difference from our results may be due to the different combination treatment in our study with other studies. From our results, especially for stroke, the combination group had a higher incident rate than the LAAC-only or LABA-only groups. More clinical evidence is required to demonstrate the safety of cardiovascular and cerebrovascular events in COPD patients treated with combination treatment.

The main biological mechanism of vascular diseases in COPD patients using LAAC or LABA is still unclear. From previous data, COPD is known as chronic inflammatory process including lungs, metabolic syndrome, or cardiovascular diseases.^26^ The potential role of inflammatory processes plays an important role in mediating systemic cardiovascular disease in COPD.^27,28^ Previous researches showed that LAAC or LAAB-effected inflammatory cytokines might be the mechanism of high risk of vascular disease.^29,30^ Combination of LAAC/LABA or LABA/ICS may enhance the level of affected inflammatory cytokines. Otherwise, microRNAs (miRs) or regulatory noncoding RNAs (ncRNAs) represent critical regulators of cardiovascular or cerebrovascular function and play important roles in basic regulatory mechanisms of cells (including inflammation).^31–33^ Furthermore, miRs are involved in the pathogenesis of lung diseases such as asthma and COPD.^34^ In 2010, Sato et al^35^ had reported that miR-146 expression in COPD patients after stimulation by proinflammatory cytokines is less than its rate compared with patients without COPD. From current evidence, miRs are dysregulated in cardiac disease and have emerged as promising therapeutic targets.^33^ There is limited information about LAAC, LABA, or combination on the influence level of miRNA expression; more direct evidence is required to conduct the mechanism and emerging functional role of miRNA in COPD patients treated with bronchodilators, especially in different treatment strategies.

From a review, Matarese and Santulli^36^ indicated that angiogenesis is a central component of COPD, and angiogenesis is also an important component of pathophysiology in cancer and other disorders. Angiogenesis requires a tightly coordinated guidance from a variety of regulators; vascular endothelial growth factor (VEGF) is one of most important players. VEGF has higher expression in COPD patients and smokers than the general population, and this expression is associated with enlargement of arterial wall and hypoxia.^36^ Otherwise, recent evidence suggests that VEGF also acts directly on neuronal progenitor cells to produce neurogenic effect.^37^ There were previous studies which showed that combination (ICS/LABA) may enhance decreasing expression of VEGF.^38–40^ Therefore, combination treatments of LAAC/LABA or LABA/ICS may therefore be associated with enhancing the clinical effect of COPD control, and may also result in decreasing neurogenic effects in the combination group than in the single-treatment groups by decreasing VEGF expression in the brain and the lungs. However, currently unavailable evidence of LAAC or LAAB, or combination exerts a central or systemic angiogenesis effect or VEGF expression in COPD patients; further research is warranted to examine the existence of this mechanism. Otherwise, anticholinergic properties might be the mechanism causing vascular diseases in COPD patients using LAAC. Muscarinic receptors and cholinergic innervation was found in human cerebral arteries.^41^ LAAC or LAAC/LABA combination may block the relaxation of smooth muscle of arteries in brain to change the cerebral blood flow.

This study has some limitations which merit discussion. First, patient demographic information, such as smoking history and smoking status, body weight, family history of stroke, and COPD severity, was not available from the NHIRD, which could cause some bias due to lack of major cardiovascular risk factors. Furthermore, no information was available regarding lung function, laboratory data, information of cardiac examination, image of the brain or cardiovascular, which may be a potential confounder. Second, this is a retrospective cohort study, selection bias, misclassification, or information bias may occur in this study by using large health insurance database. Patients using LABA, LAAC, or combination over 1 year were included in the analysis, which could cause selection bias for patients who are using for less than 1 year or who are intermittent users. Hospital and outpatient visit claim data were used to determine the diagnoses, medical treatment, and duration of treatment. Moreover, the possibility of selection bias and misclassification cannot be completely ruled out because some patients may not have used their prescriptions. Otherwise, accuracy of the information used in this study, such as for prescribed drugs and comorbidities, has not been validated, which could also leave room for information bias. Third, the patients in our sample were Han Chinese; generalization of the results to other ethnic or racial groups must therefore be made with caution. Lastly, because of prevalent medication use of LABA or LAAC, or combination, analysis of these medications on the vascular risk was limited by the population and sample size. Therefore, future studies are recommended to recruit a larger COPD population to further investigate the correlation between bronchodilators and safety of vascular disease.

## CONCLUSIONS

This study retrospectively used a large health insurance database and Cox hazard modeling to evaluate the risk of stroke and cardiovascular diseases in 3 different COPD treatment groups (LAAC only, LABA only, or a combination of LAAC/LABA or LABA/ICS). Our results suggest that the combination treatment may increase stroke risk compared with the single-drug treatment. COPD patients treated clinically with a combination of LAAC/LABA or LABA/ICS should be monitored more for the hazard of stroke and cardiovascular events.
