# Long-acting Inhaled Bronchodilator and Risk of Vascular Events in Patients With Chronic Obstructive Pulmonary Disease in Taiwan Population: Erratum

**DOI:** 10.1097/01.md.0000481354.70978.fe

**Published:** 2016-03-03

In the article “Long-acting Inhaled Bronchodilator and Risk of Vascular Events in Patients With Chronic Obstructive Pulmonary Disease in Taiwan Population”,^1^ which appeared in Volume 94, Issue 51 of *Medicine*, Dr. Lin's name was originally spelled incorrectly. The article has since been corrected online.
